# Circuit Parameters of a Receiver Coil Using a Wiegand Sensor for Wireless Power Transmission

**DOI:** 10.3390/s19122710

**Published:** 2019-06-16

**Authors:** Katsuki Takahashi, Tsutomu Yamada, Yasushi Takemura

**Affiliations:** Electrical and Computer Engineering, Yokohama National University, Yokohama 240-8501, Japan; kono-katsuki-rj@ynu.jp (K.T.); yamada@ynu.ac.jp (T.Y.)

**Keywords:** wiegand sensor, magnetization reversal, large Barkhausen jump, FeCoV wire

## Abstract

We previously demonstrated an efficient method of wireless power transmission using a Wiegand sensor for the application in implantable medical devices. The Wiegand sensor has an advantage in inducing sharp pulse voltage independent of the drive frequency. A down-sized receiver coil for wireless power transmission within blood vessels has been prepared, which enables medical treatment on any part of a human body. In order to develop practical applications of the Wiegand sensor as implantable medical devices, the circuit design is important. The circuit parameters in the circuit model of the Wiegand sensor must be clearly identified. However, a fast reversal of magnetization of the magnetic wire used in the Wiegand sensor, known as a large Barkhausen jump, and the induced nonlinear pulse signal make the inductance of the receiver coil time-dependent and inconsistent as conventionally considered in circuit analysis. In this study, the voltage and current responses of a wire-core coil are analyzed, and the time-dependent inductance is determined. The results showed that the inductance depends on the magnetization state of the wire, which can be negative during the fast reversal of magnetization.

## 1. Introduction

Implantable medical devices, such as capsule endoscopy, are operated within the human body. In future, multi-functionalized microrobots are expected to be inserted into a patient’s body through the blood vessels. To this end, downsizing to a diameter of blood vessels and prolonging the operation of these implantable medical devices are necessary. However, challenges remain as long as they are operated using cell batteries. One outstanding technology that may substitute the batteries is wireless power transmission using inductive coupling. It is, however, necessary to limit the intensity and frequency of the magnetic field applied on a human body, which may cause heat and stimulus effects. These factors become more serious as the drive frequency increases [1]. The International Commission on Non-Ionizing Radiation Protection (ICNIRP) suggests limiting the exposure of magnetic and electric fields for general and occupational situations [2,3]. Therefore, the drive frequency must be set at a lower value, below 10 kHz, even though an inductive coupling system is directly dependent on the frequency. A lower drive frequency would result in reduced voltage and electrical power. In this work, as a practical solution, a twisted FeCoV wire in the Wiegand sensor is used for the receiver coil, and a frequency-independent pulse output induced in the coil is used for the power supply. We have shown that effective wireless power transmission is achieved using the FeCoV wire, particularly for the excitation frequency range lower than 10 kHz [4]. This study proposes a circuit model of the Wiegand sensor used in wireless power transmission and investigates the circuit parameters.

Implantable medical devices for hyperthermia are good examples for converting an externally supplied alternating magnetic field to energy for wireless power transmission. Hyperthermia is a thermal therapy for cancer. It elevates the body temperature to above 42.5 °C to kill cancer cells selectively. A ferrite core with high permeability is used for hyperthermia implants [5,6], similar to that used as a conventional coil-core for the receiver coil in the inductive coupling for wireless power transmission. We have recently proposed the use of a FeCoV wire instead of ferrite as the coil core material for inductive coupling. The voltage induced in the receiver coil with a ferrite core is essentially linear to the frequency of the applied alternating field. Compared to the conventional method using a ferrite core having high permeability, such as MnZn, the receiver coil with the FeCoV wire induces a pulse voltage independent of the applied field frequency and generates electrical power effectively in the lower frequency range below 10 kHz [4]. In addition to its use as the power source of medical devices, the Wiegand sensor can also be used as an energy-harvesting module and power source for the battery-less operation of electronic devices. [7,8].

In these applications of the Wiegand sensor, electrical circuits, e.g., a rectifying circuit, charging capacitor, and load circuit, are connected to the sensor. Then the internal circuit parameters in the equivalent circuit of the Wiegand sensor, especially the inductance of the receiver coil, should be taken into consideration. Although the application of the Wiegand sensor for the battery-less operation of a rotary encoder [9] is an attractive topic, particularly from the point of view of the industry, only few articles on the Wiegand sensor have been published [10,11] other than those by our group [7,8,12,13]. As Saggini et al. reported for the circuit analysis of the Wiegand sensor for low-power energy harvesting solutions [10], it is significant to study the circuit parameters for applications of the Wiegand sensor.

The motivation for this work is that circuit design and simulation are necessary for developing the applications of the Wiegand sensor. To achieve that, an equivalent circuit with circuit parameters should be determined. The objective of this study is to derive the circuit parameter, particularly the inductance of the receiver coil, which changes depending on the magnetization state of the wire inside. In this study, the circuit parameters, including the time-dependent inductance of the receiver coil (pick-up coil) in the Wiegand sensor, are successfully determined by measuring the waveforms of the current-voltage characteristics. We found that the inductance changed periodically followed by an alternating excitation field depending on the magnetization state of the wire and that it can be negative during the fast reversal of magnetization. This result provides a significant concept of inductance in the Wiegand sensor as this time-dependent inductance is required to perform an accurate circuit design and analysis for applications of the Wiegand sensor.

## 2. Materials and Method

The Wiegand sensor used in this study is based on the Wiegand effect, proposed by J. R. Wiegand in 1974 [14]. Originally, this behavior was observed in magnetic wires of NiFe alloys [8]. Vicalloy, having a typical composition of Fe_0.4_Co_0.5_V_0.1_, is known to be one of the most suitable materials that exhibit this effect [15,16,17,18]. In addition, a large Barkhausen jump in a magnetically bistable FeSiB amorphous wire is also observed [19,20].

**Figure 1:**
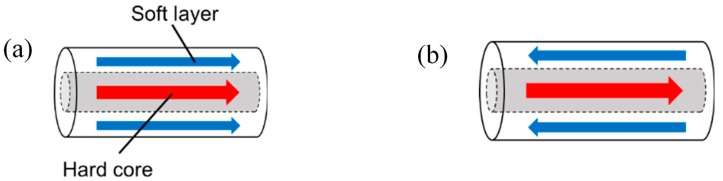
Two states of magnetization of the FeCoV wire: (**a**) parallel state and (**b**) antiparallel state.

**Figure 2:**
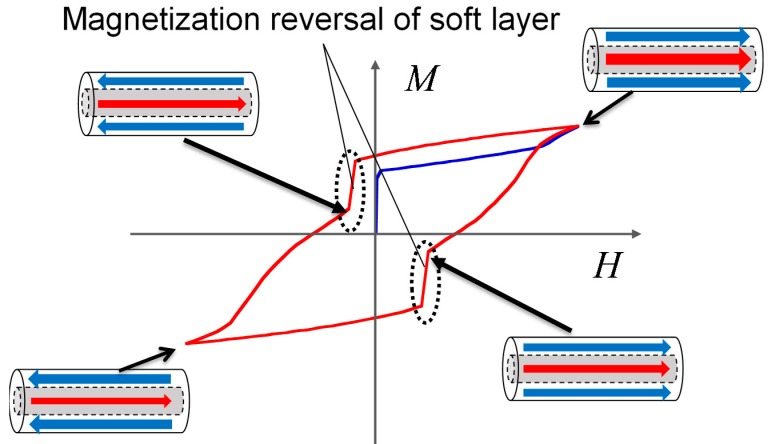
Initial magnetization curve (blue) and minor hysteresis loop (red) of the FeCoV wire.

In our experiments, a twisted FeCoV wire is used. This wire has the same magnetic properties as those reported in previous publications [7,12]. The wire exhibits two layers with different magnetic properties in its core (center) and surface regions. In order to achieve an optimum magnetic property of the wire to yield the Wiegand effect, annealing and torsion stress are applied to the wire. The magnetic properties of twisted FeCoV wires depending on the conditions of annealing and torsion stress have been previously reported in detail. [17]. Torsion stress is first applied to the wire during its preparation. When the stress is released, the outer layer near the surface becomes magnetically soft, and the inner core remains magnetically hard. As shown in Figure 1, the magnetization alignment of the soft layer and the hard core can either be in a parallel or antiparallel state. The magnetization reversal of the soft layer is accompanied by a large Barkhausen jump. This fast magnetization reversal independent of the changing rate of the applied field induces a pulse voltage in the pick-up coil wound around the wire. The voltage is independent of the frequency of the external magnetic field. For its use in wireless power transmission, an alternating magnetic field switches the magnetization of the soft layer and generates a series of pulse voltages in the pick-up coil. Thus, it assumes both the states in turns. This process appears as a nonlinear minor hysteresis loop, as shown in Figure 2.

**Figure 3:**
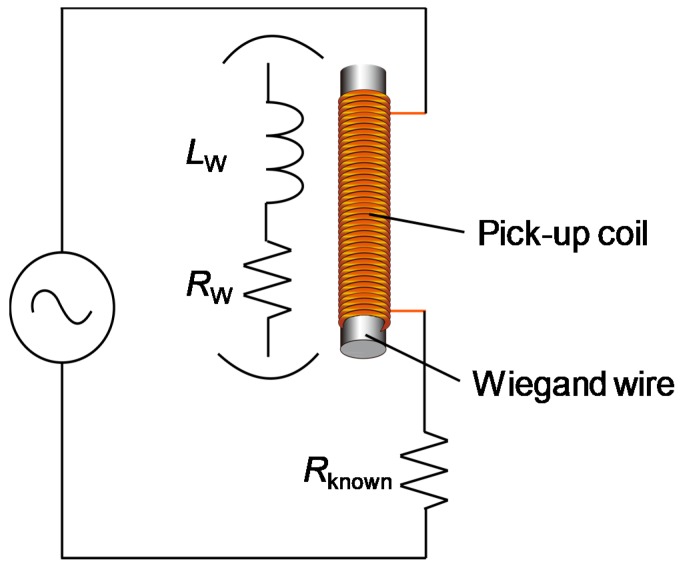
Configuration for the measurement of the Wiegand sensor connected with the load resistor and power source with an alternating current. The Wiegand sensor is described by its circuit model composed of the inductance and resistor of the pick-up coil as circuit parameters.

A twisted FeCoV wire of 11 mm length and a 0.25 mm diameter was used in this work. It had a coercive field of 2 mT in the soft layer and 8 mT in the hard core. The saturation magnetization of the sample was *M*_s_ = 1.78 T. The details of the magnetization properties of full and minor magnetization curves have been reported [7]. The circuit model of the coil in the Wiegand sensor can be simply represented using an internal resistance and an inductance, as shown in Figure 3. A similar circuit model for the Wiegand sensor was reported recently [10], but the purpose of this study is to analyze the time-dependent current-voltage characteristics under the excitation by an alternating magnetic field in a sinusoidal waveform and to derive the circuit parameters. A 3000-turn coil is wound around the FeCoV wire; this pick-up coil had a diameter of 2 mm and a length of 9 mm. This combination of the coil and the wire allows for medical treatments inside narrow parts of the human body (such as blood vessels) and the supply of enough DC electric power into the connected module [4]. The internal resistance of the core coil was 175 Ω, which was measured at a frequency range of 1–100 kHz using an LCR meter (HIOKI IM3536). For the inductance measurement, the coil was directly connected to a sinusoidal signal generator and a known resistance (50 Ω) for monitoring the current signal, as shown in Figure 3. A sinusoidal current flowed into the coil to generate an AC magnetic field, which was then applied to the core (wire). The signal frequency was set to 1 kHz, and the voltage response of the coil and that of the current through the resistor were measured under varying conditions–allowing the calculation of the inductance. Although the objective of this study is to develop implantable medical devices using the Wiegand sensor, all measurements were performed in normal atmosphere at room temperature (20–25 °C).

## 3. Results and Discussion

### 3.1. Magnetic Properties of the Twisted Fecov Wire

**Figure 4:**
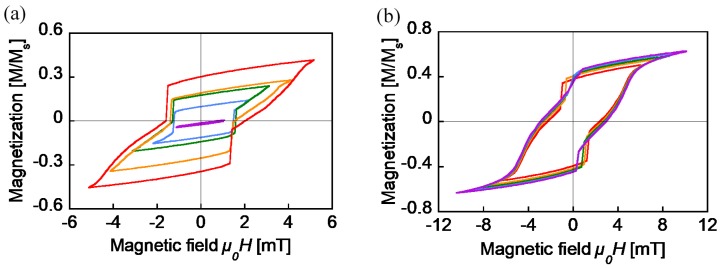
DC minor loops of the FeCoV wire (0.25 mm diameter and 11 mm length). The applied field intensities are (**a**) *μ_0_H* = 1–5 mT and (**b**) *μ_0_H* = 6–10 mT.

To determine the magnetic properties of the twisted FeCoV wire, DC (static) minor loops were traced with an applied alternating magnetic field of *μ_0_H_ext_* from 1 mT to 5 mT, as shown in Figure 4a. Similar measurements from 6 mT to 10 mT are shown in Figure 4b. These were measured by a vibrating sample magnetometer (VSM). Except for the loop of the 1 mT excitation, two fast magnetization reversals accompanied with a large Barkhausen jump were observed. This indicates a magnetization transition from a parallel to an antiparallel state, and vice versa, which makes the curve nonlinear, as shown in Figure 2. The magnetization process after the reversal of the soft layer is also important. The higher the external field, the slower the increase of the magnetization. Owing to the larger coercive field of the hard core as *μ_0_H_ext_* approaches 8 mT, almost all the magnetization in the wire followed its direction and finally saturated. This indicates that the permeability of the FeCoV wire is not static; rather, its value reduces as it reaches saturation. These minor loops are a result of the magnetization of the wire due to the external magnetic field. However, the same tendency is expected in the inductance of the wire-core coil.

### 3.2. Inductance of the Wire-Core Coil

#### 3.2.1. Under the Condition that the Wire Has No Reversal Process

**Figure 5:**
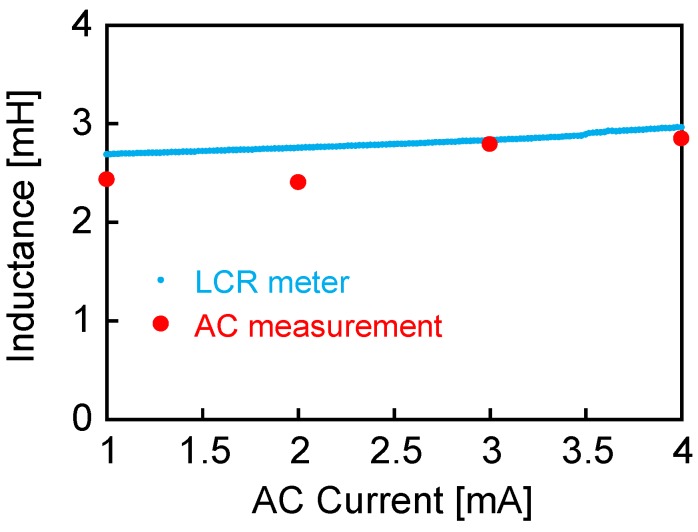
Inductance value under an AC current of small amplitude (without introducing any large Barkhausen jumps) applied to the circuit shown in Figure 3.

The inductance of the wire-core coil was studied using the circuit shown in Figure 3. Under the condition that the current amplitude is small, in other words, the intensity of the AC magnetic field in the coil is less than 2 mT, the wire was excited, with no occurrence of large Barkhausen jumps. Both current and voltage responses are sine waves, and the inductance is calculated simply by the differences between the two signals. Figure 5 shows the calculated value of the inductance. The current amplitude was set to less than 4 mA, while the field intensity from the coil was 2 mT when the amplitude reached 5 mA. The magnetization of the wire might trace a linear curve, similar to the *μ_0_H_ext_* = 1 mT loop, as shown in Figure 4a. The inductance had a fixed value at around 2.5–2.7 mH, which increased from that of the air core coil of 1.1 mH. This inductance calculated under the AC current measurement agrees well with that measured by the LCR meter, as shown in Figure 5.

#### 3.2.2. Under the Condition that the Wire is Oriented to the Easy Axis

**Figure 6:**
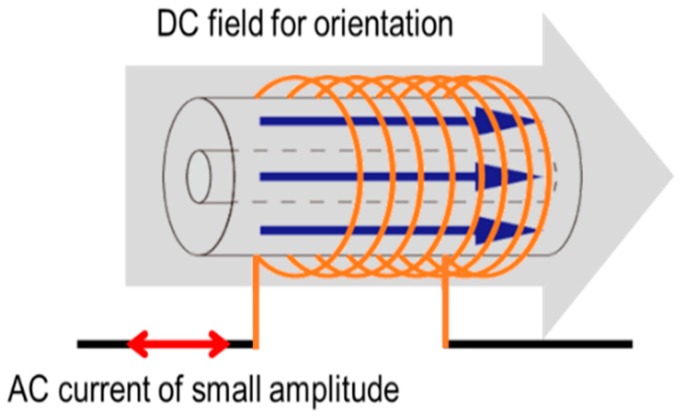
Excitation of the wire by AC current under DC bias field to orient the magnetization.

In practical applications, the magnetization process of the FeCoV wire normally forms a nonlinear hysteresis. Considering the current flowing into the coil and the change in the magnetic flux in the wire, the inductance is variable depending on the instantaneous state of the magnetization of the wire. Here, a DC magnetic field was applied as a bias field in the longitudinal direction of the wire, as shown in Figure 6. The magnetization value should depend on the intensity of this DC field. When an AC magnetic field of 1 kHz (with amplitude less than the threshold to obtain large Barkhausen jumps caused by the coil current) was applied simultaneously with the DC bias field, a linear hysteresis loop was observed. The inductance should be equivalent to the loop’s gradient, which depends on the state of magnetization. From the induced voltage and the current signals, the inductance was calculated as described in the previous section.

**Figure 7:**
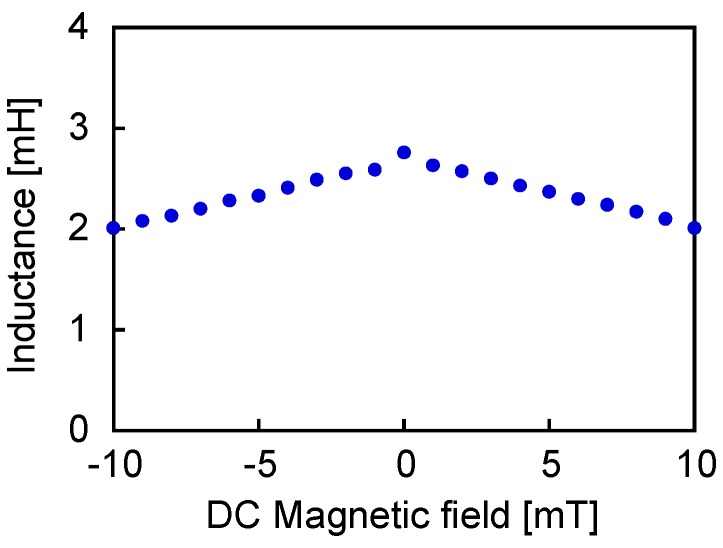
Inductance of the coil in the Wiegand sensor depending on DC bias field.

Figure 7 shows the calculated value of inductance, indicated as a function of the DC field intensity. Here, the AC current throughout the coil was set to 1 mA amplitude. The inductance was reduced by applying the DC field to the wire, regardless of the direction. This result proves that the inductance of the coil in the Wiegand sensor has a rather nonstatic value, which depends on the state of the magnetization of the wire.

#### 3.2.3. Under the Condition that the Wire Undergoes a Reversal Process

**Figure 8:**
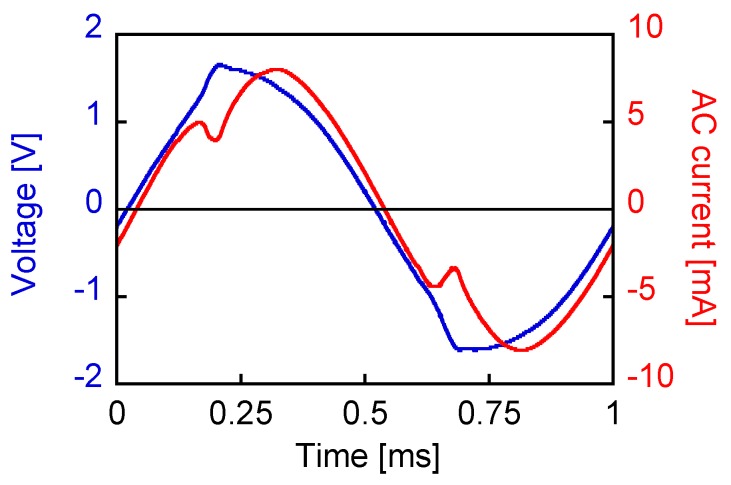
Voltage and current wave when an AC current of 8 mA amplitude was applied (1 kHz).

**Figure 9:**
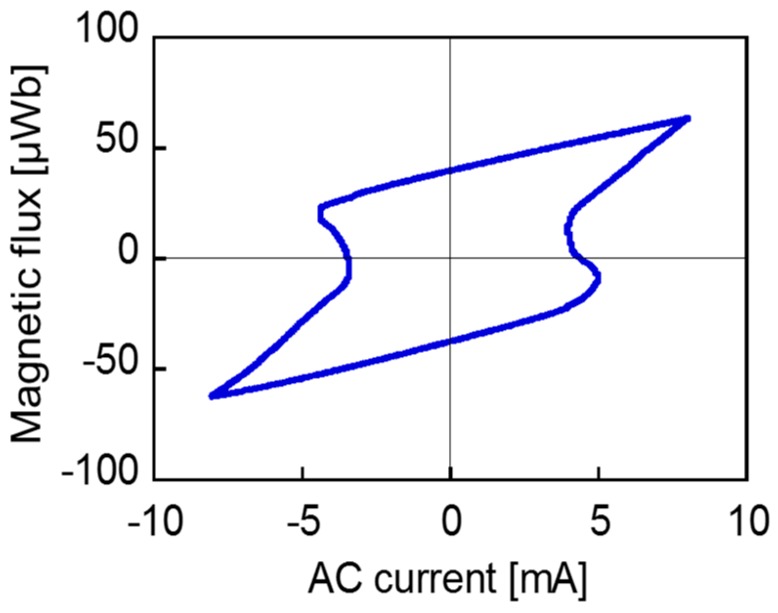
*Φ*_w_-*I*_w_ loop derived from the coil voltage and current wave in Figure 8.

Figure 8 shows the waveforms of voltage across the coil and current (with a 8 mA amplitude) through it. In this case, an AC magnetic field of 3 mT was applied to the wire, which induced a large Barkhausen jump, and the voltage waveform was distorted. The Wiegand pulse was superposed on to the induced voltage in a sine wave. However, this cannot be clearly observed. This is because the amplitude of the induced voltage is sufficiently large to dominate the composite wave. In addition, the current wave is nonlinear at the moment of pulse generation. Self-induction occurs in the pick-up coil due to the fast transient of the magnetic flux inside the wire. This opposes the current and tries to prevent the change in the magnetic flux. This phenomenon and the two waves of the voltage and current make it difficult to analyze the phase difference between the voltage and current in the conventional method for an alternating current circuit. Figure 9 shows the *Φ*_w_–*I*_w_ curve, processed and calculated from the two waveforms above, where *Φ*_w_ and *I*_w_ denote the magnetic flux and AC current of the wire-core coil, respectively. To derive this curve, the following steps were undertaken. The waveshape across the pick-up coil contained both components: internal resistance and inductance. The product of the current wave and the value of the internal resistance were subtracted from the original wave in order to remove its components. Then, the remainder component (i.e., the inductance-originated wave) was integrated with time, which provides the magnetic flux, Φw. The horizontal axis (i.e., the instantaneous current) and the vertical axis, Φw, formed a hysteresis-like curve, as shown in Figure 9. The loop became narrow at the two-turning points of the magnetization reversal due to the self-induction of the pick-up coil that opposed the flow of current.

**Figure 10:**
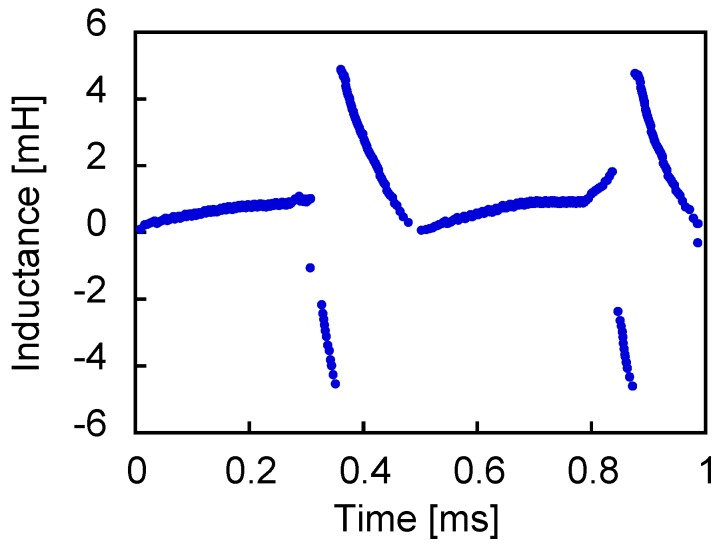
Inductance variability in one cycle from the *Φ*_w_-*I*_w_ loop (1 kHz).

With the *Φ*_w_ = *LI*_w_ relationship, the gradient of the *Φ*_w_–*I*_w_ curve in each time step was regarded as the value of the inductance. Figure 10 shows its transient in one cycle when a sinusoidal voltage of 1 kHz was applied to the circuit. The self-induction caused in the pick-up coil by large Barkhausen jumps and the nonlinear voltage wave by the Wiegand pulse varied the inductance sharply. Then, it assumed a negative value.

**Figure 11:**
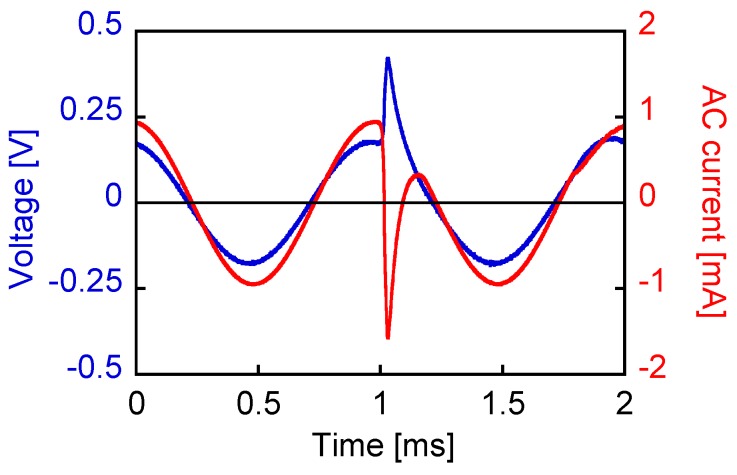
Voltage and current waves when an AC current of 1 mA amplitude is applied. A large Barkhausen jump is caused by the magnetic field of a slow-moving magnet having dimensions of 3 × 3 × 5 mm^3^.

**Figure 12:**
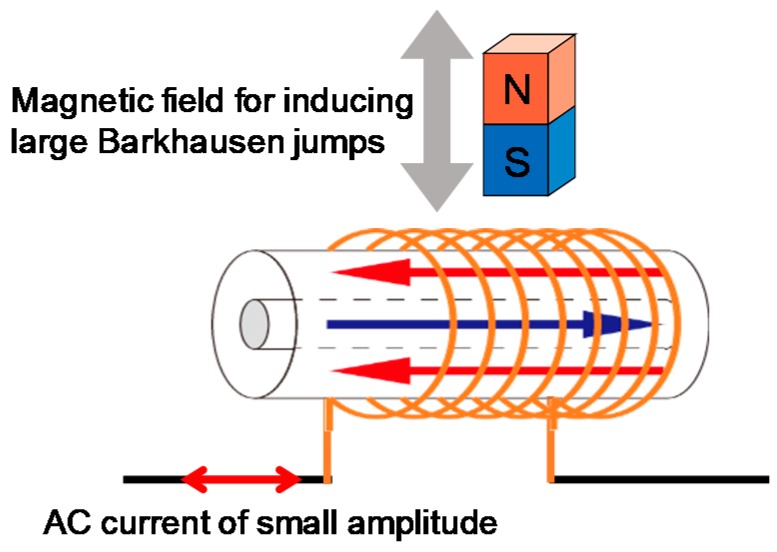
Application of an AC current of small amplitude where a large Barkhausen jump occurs due to the independent magnetic field of the slow-moving magnet shown in the circuit of Figure 3.

Figure 11 shows the voltage and current waveshapes when an AC current of 1 mA amplitude at a frequency of 1 kHz was applied. The wire was excited sufficiently by approaching a magnet of dimensions 3 × 3 × 5 mm^3^. The movement was extremely slow so as to minimize the changing rate of the applied field. This pulse-inducing magnetic field was separated from the circuit in this measurement, as shown in Figure 12. Thus, the pulse component in the voltage was observed more clearly than the waveshape shown in Figure 8. The nonlinear effect was also observed in the current wave. The large Barkhausen jump opposed the current flow, resulting in the change and nonlinear behavior of the AC current and voltage.

## 4. Conclusions

In this work, equivalent circuit parameters of resistance and inductance of the wire-core coil used in Wiegand sensor were measured using an LCR meter and current-voltage characteristics under the excitation of alternating current. The inductance of the coil strictly reflected the magnetization state of the wire. At the moment when a large Barkhausen jump occurred, the coil underwent self-induction due to the fast propagation of the magnetic flux inside the wire. Consequently, the calculated inductance became negative. In order to optimize the performance of inductive coupling using the Wiegand sensor, the determination of internal parameters in the circuit design is required. The obtained experimental results of the nonlinear characteristics of the current and voltage and the time-dependent inductance of the Wiegand sensor are significant in developing applications of the Wiegand sensor.
